# Assessment of the Crystallization Process of CaO–Al_2_O_3_–SiO_2_ Glass Probed with Tb^3+^ Luminescence

**DOI:** 10.1021/acs.inorgchem.2c01950

**Published:** 2022-07-13

**Authors:** Shingo Machida, Takuma Yamaguchi, Naoki Emori, Ken-ichi Katsumata, Kei Maeda, Atsuo Yasumori

**Affiliations:** Department of Material Science and Technology, Faculty of Advanced Engineering, Tokyo University of Science, 6-3-1 Niijuku, Katsushika-ku, Tokyo 125-8585, Japan

## Abstract

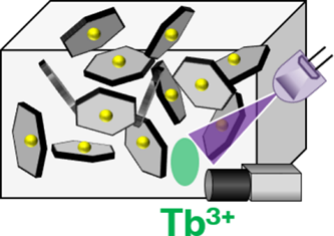

The ratio of the intensity of Tb^3+^ fluorescence at 543 nm because of an electric dipole transition (^5^D_4_–^7^F_5_) relative to that at 437 nm due to a magnetic dipole transition (^5^D_3_–^7^F_4_) was determined to be proportional to the amount of metastable CaAl_2_Si_2_O_8_ crystals precipitated in CaO–Al_2_O_3_–SiO_2_ glass. The present results indicate that Tb^3+^ luminescence can be used as a probe to evaluate the crystallization of glass.

## Abstract 2

The ratio of the intensity of Tb^3+^ fluorescence at 543 nm due to an electric dipole transition (^5^D_4_−^7^F_5_) to that at 437 nm because of magnetic dipole transition (^5^D_3_−^7^F_4_) was used to probe the precipitation of metastable CaAl_2_Si_2_O_8_ crystals in CaO−Al_2_O_3_−SiO_2_ glass.

## Introduction

Glass-ceramics (GCs)^1−5^ are composite materials produced by precipitating crystalline phases in glassy phases by heat treatment. Following the initial reports of these materials,^1^ their crystalline microstructures were widely investigated as a means of developing practical applications.^3−5^ It is thus important to obtain information concerning crystalline phases embedded in glassy phases, although characteristics of crystals can be overlapped by those of glasses. In this case that the compositions of the crystalline and glassy phases can be close to one another, these two phases cannot be separately assessed. X-ray diffraction (XRD) offers one approach to estimating the degree of crystallinity in GCs based on the strong reflections to crystalline phases. Among the crystals that can precipitate in glass, layered crystals such as micas comprise stacked inorganic layers, and the stacking order and number in these materials vary with the types of ions between the layers as well as the lateral sizes of the layers and the degree of crystallinity.^6−9^ The intensity, width, and position of diffraction lines in the XRD patterns will change with variations in the stacking direction of these layers. A wide range of different patterns can therefore be obtained from layered crystals in GCs even if these layered inorganic solids are present in constant amounts in glasses. In addition, it can be difficult to extract information of crystals in XRD patterns if the crystal content of the GC is extremely low. For these reasons, it is helpful to assess changes in the glassy phase as the amount of the crystalline phase increases in relatively lower crystal fraction ranges, because the glassy phase is present in a larger proportion in GCs in initial stages of glass crystallization. In particular, it would be beneficial to develop means of tracking decreases in the volume fraction of the glassy phase induced by crystallization based on the concentration of probe ions added to the glassy phase.

Herein, we report the effect of the crystallization of CaO–Al_2_O_3_–SiO_2_ (CAS) glass on the luminescence behavior of Tb^3+^ contained in the glass. The structure and composition of glass have previously been probed based on assessing the luminescence of rare-earth ions, which varies with the concentration of such ions.^10−19^ Typically, the more intense fluorescence from these ions, resulting from electric dipole transitions, is perturbed by changes in the local structure, whereas the fluorescence due to magnetic dipole transitions is unaffected such that the ratio of the intensities of these fluorescence outputs is modified. CAS glass precipitated with micron-sized particles of metastable CaAl_2_Si_2_O_8_, a layered aluminosilicate with a hexagonal plate-like morphology (referred to herein as CAS-H), displays relatively high transparency compared with GCs precipitated with micas.^20−24^ CAS-H materials also exhibit a low crystal fraction.^20−24^ The crystals that are formed in glass are precipitated using metallic Mo particles as nucleation agents based on the reduction of MoO_3_ by C during the glass melting stage.^20−24^ Therefore, in the present study, variations in the Tb^3+^ fluorescence intensity ratio related to the precipitation of metastable CaAl_2_Si_2_O_8_ in CAS glass were investigated. Prior to these trials, Eu^3+^-doped CAS glass was also prepared and crystallized. Eu^3+^ ions were used because these ions are widely employed for the purpose of various materials^10−13^ including glass-ceramics precipitated with micas^25^ and because their valence state is easily changed under a reductive atmosphere.^26^

## Experimental Section

In this study, 50 g of CAS glass specimens was prepared using a conventional laboratory-scale melting method based on heating the raw materials at 1550 °C for 1 h under air in an alumina crucible.^22,24^ All raw materials were reagent grade and were obtained from Wako Pure Chemical or Kojundo Chemical Laboratory Co., Ltd. The nominal composition of the glass was 25CaO–20Al_2_O_3_–55SiO_2_ (wt %) with 0.05 wt % MoO_3_ and 0.40 wt % C^22,24^ and 0.37, 0.74, 1.06, 1.48, or 2.96 wt % Tb_2_O_3_. Herein, each glass is referred to as CAS-*x*Tb, where *x* represents the Tb^3+^ content. It should be noted that these Tb^3+^ concentrations in the specimens corresponded to 0.15, 0.30, 0.43, 0.60, and 0.90 mol %, while the nominal glass composition was 28.6CaO–12.6Al_2_O_3_–58.8SiO_2_ with 0.02MoO_3_ and 2.1C in mol%. The CAS-0.74Tb and -1.48Tb specimens were crystallized by heating at 950 °C for 2, 4, 6, or 8 h to give products referred to herein as CAS-0.74 or 1.48Tb-*y*, where *y* represents the heat treatment time. For comparison, a glass specimen containing 0.74 wt % Tb_2_O_3_ but without MoO_3_ and C was also prepared (Mo-Free-CAS-0.74Tb) and was heat-treated at 1000 °C for 9 h. Additionally, a glass with the composition of 27CaO–13Al_2_O_3_–60SiO_2_ (wt %) with 0.74 wt % Tb_2_O_3_ was fabricated (Mo-Free-Al-low-CAS-0.74Tb). The heating and cooling rates applied to promote crystallization during the present work were similar to those employed in our previous studies.^22,24^ It should be noted that a 0 h heat treatment indicates that the glass did not undergo heat treatment and also was not subjected to elevated temperatures and subsequent cooling. Prior to these experiments, glass specimens with a composition 28.6CaO–12.6Al_2_O_3_–58.8SiO_2_ with 0.02MoO_3_ and 2.1C (mol %) with and without 0.60 mol % Eu_2_O_3_ were also prepared and crystallized at 1050 °C for 2 h in a similar fashion to the Tb^3+^-doped glass specimens. The glass specimen with Eu_2_O_3_ is denoted herein as CAS-Eu, and the glass specimen without Eu_2_O_3_ is the same as the CAS-H (see Introduction). Each glass specimen was cut and polished to remove the surface layer and to provide specimens of an appropriately consistent size for subsequent analyses.

The crystal phases and microstructures of the glass were characterized by XRD and scanning electron microscopy (SEM). The crystal volume fractions (vol %) in the products were roughly estimated using binarized SEM images. Fluorescence spectra of the glass specimens before and after crystallization were acquired with excitation at 376 nm for Tb^3+^-doped glass specimens and at 393 nm for CAS-Eu specimens, averaging nine scans for each spectrum. Concerning the glass specimens containing Tb^3+^, the ratio of the intensity of the green Tb^3+^ luminescence (due to the ^5^D_4_–^7^F_5_ electric dipole transition) at 543 nm to that of the blue Tb^3+^ luminescence (due to the ^5^D_3_–^7^F_4_ magnetic dipole transition) at 437 nm was determined. The resulting value (intensity (^5^D_3_–^7^F_4_)/intensity (^5^D_4_–^7^F_5_))^14−19^ is denoted herein as the B/G ratio for simplicity. Prior to the estimation, the spectra were normalized relative to the luminescence intensity at 543 nm. It should be noted that a noise value of 2.0 × 10^–2^ resulting from the light source is included as an error bar for some of the B/G ratios in the plots presented herein.

## Results and Discussion

The parent CAS-H glass appeared black in color because of the presence of metallic Mo particles, in good agreement with a previous report,^23^ while the CAS-Eu was yellowish (Figure S1a,b). The Eu^2+^-doped glass produced in prior study also had a yellow color.^27^ The fluorescence spectrum of the CAS-Eu parent glass (Figure S1c) exhibited a broad emission band at approximately 470 nm resulting from Eu^2+^ ions.^27^ Reflections attributed to metastable CaAl_2_Si_2_O_8_^20^ were observed in the XRD pattern obtained from the CAS-H, in agreement with a previous report, which were absent from the XRD pattern generated by the CAS-Eu (Figure S2). This result suggests that Eu^3+^ ions were preferentially reduced to Eu^2+^ by carbon in the glass instead of MoO_3_ during the melting stage. For this reason, Eu ions were deemed not suitable for probing the crystallization of CAS glass in this study.

Fluorescence spectra of the glass specimens containing Tb^3+^ and the B/G ratios obtained from these materials (Figure S3, left and right) indicate that the intensity of the 543 nm emission increased with increasing Tb^3+^ content, while the B/G ratio decreased. These results are in good agreement with those reported previously, in which blue luminescence at 437 nm was quenched with increasing Tb concentration while the green luminescence at 543 nm was not.^17−19^ Because the normalized spectrum obtained from the CAS-0.37Tb was relatively noisy and the B/G ratio for the CAS-2.96Tb was relatively small (Figure S3, middle and right), the CAS-0.74Tb and -1.48Tb specimens were determined to be the best candidates for the crystallization process.

**Figure 1:**
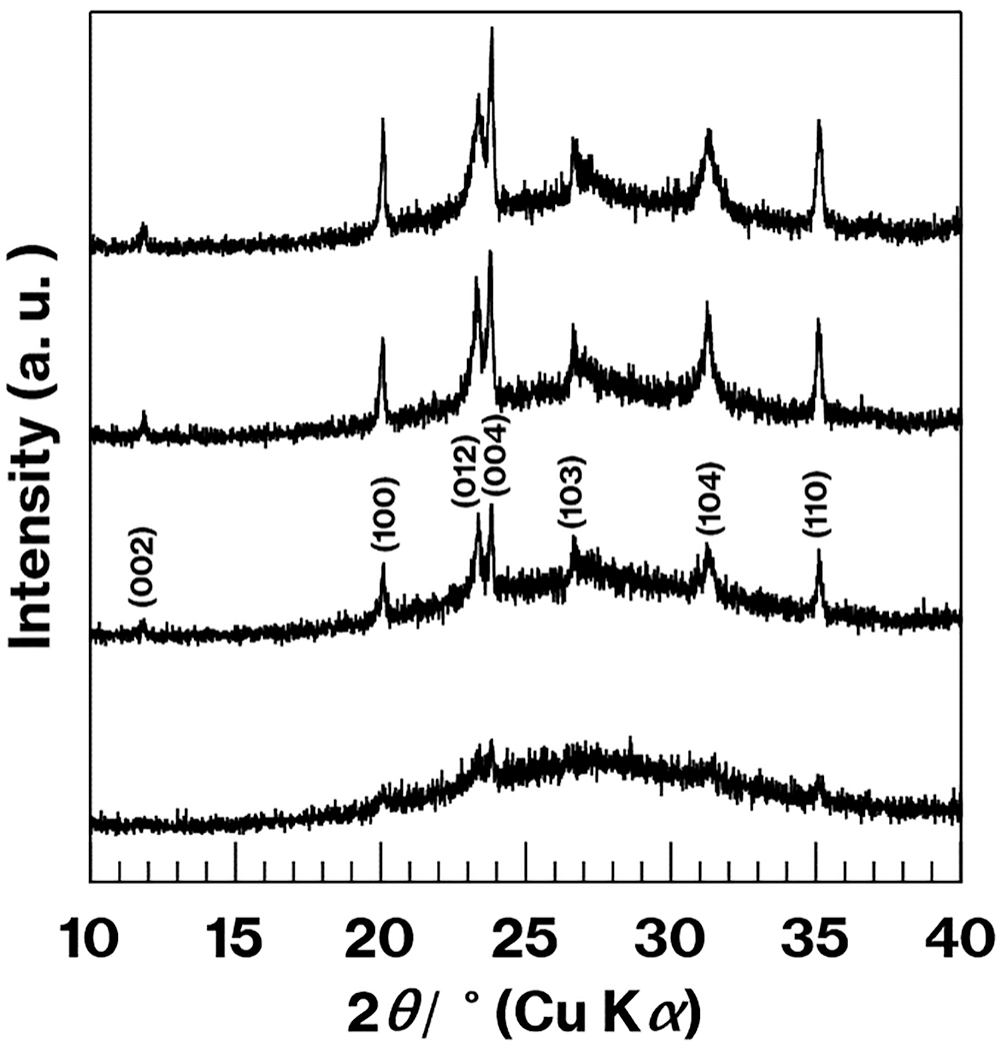
XRD patterns obtained from CAS-1.48Tb-2 h, −4 h, −6 h, and −8 h (from the bottom to the top).

**Figure 2:**
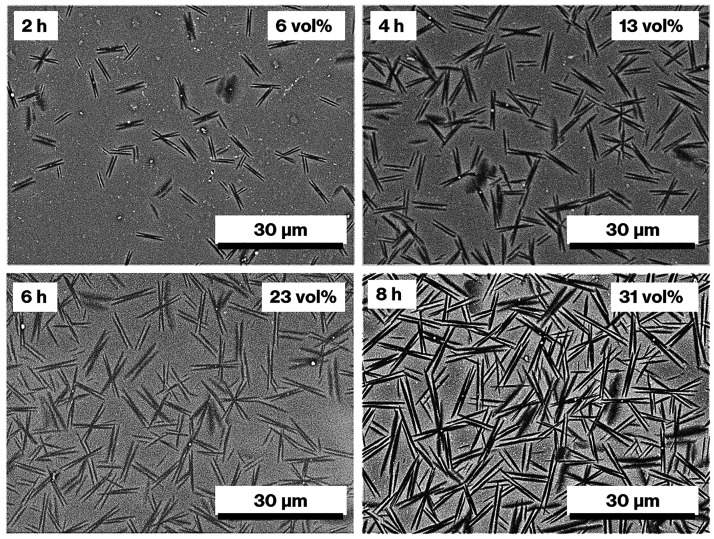
SEM images of CAS-1.48Tb-2 h, −4 h, −6 h, and −8 h. Heat treatment time and the crystal volume fraction are shown in the upper left and right of each image, respectively.

**Figure 3:**
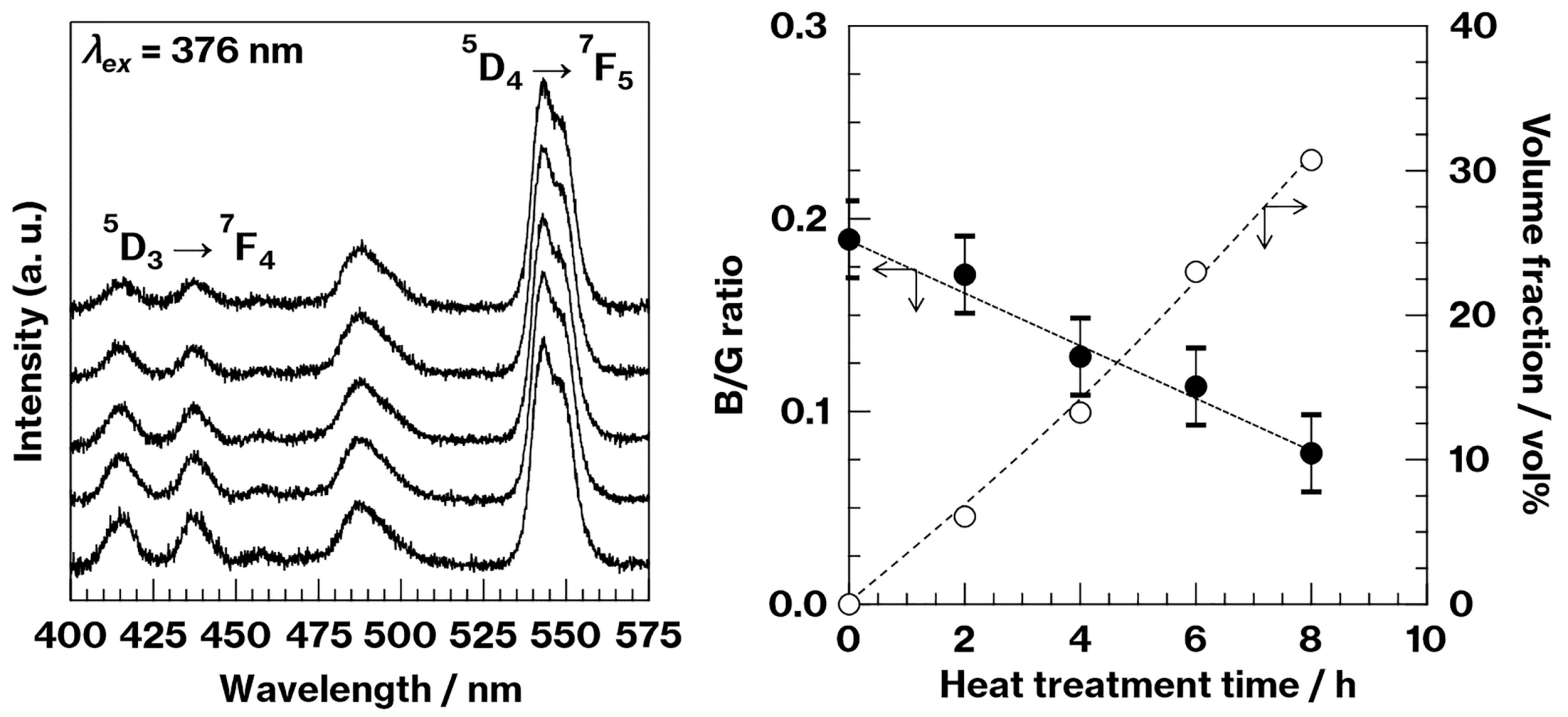
Fluorescence spectra normalized by the 543 nm luminescence intensity (left, CAS-1.48Tb, −2, −4, −6, and −8 h from the bottom to the top) and B/G ratios (filled symbols) and crystal volume fractions (empty symbols) as functions of the heat treatment time (right). The dotted and dashed lines are simply visual aids.

**Figure 4:**
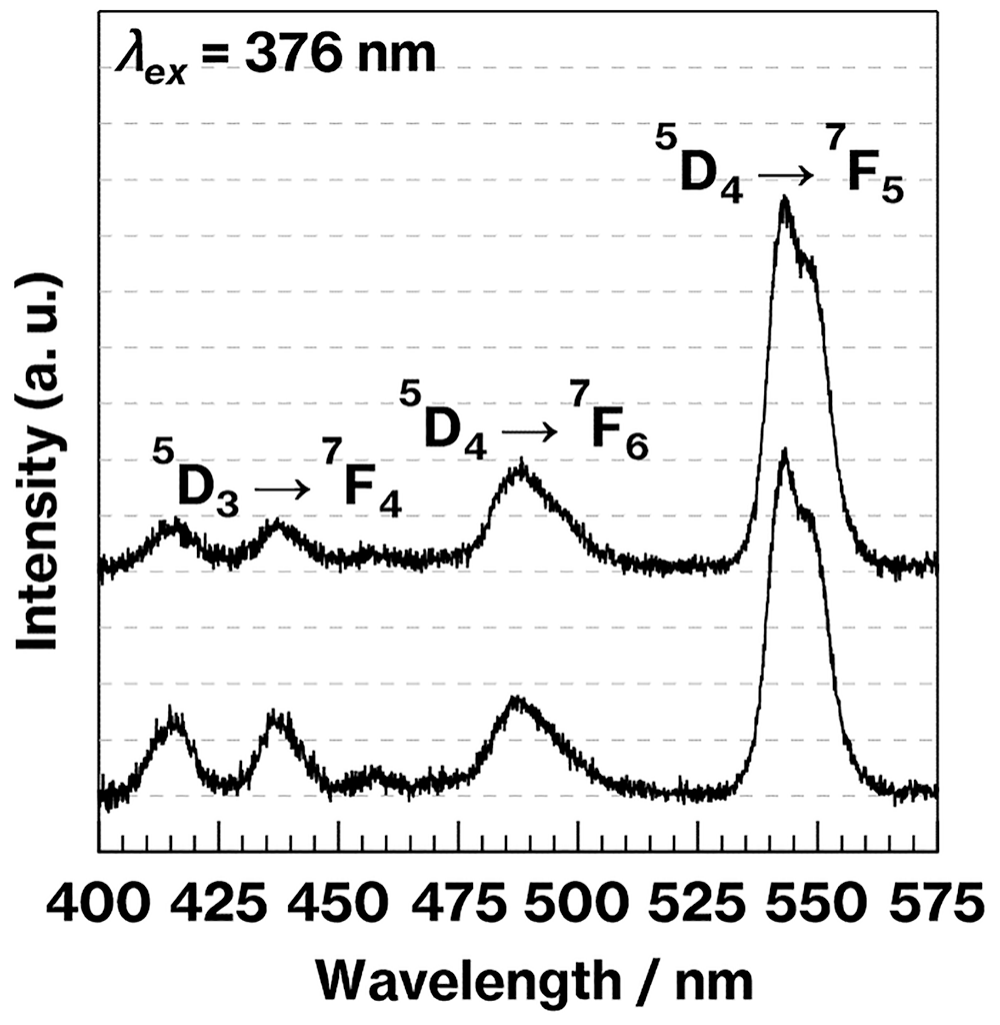
Fluorescence spectra obtained from CAS-1.48Tb and −8 h (from the bottom to the top).

XRD patterns and SEM images obtained for the various materials demonstrated an increasing amount of precipitated metastable CaAl_2_Si_2_O_8_ crystals in the CAS-0.74Tb with increasing heat treatment time (Figure S4). The XRD patterns showed an increase in the intensity of reflections^20^ because of metastable CaAl_2_Si_2_O_8_ crystals, while the SEM images confirmed that more needle-like crystals appeared in the cross-sections of the “house of cards” structure formed by the hexagonal plate-like particles in the metastable CaAl_2_Si_2_O_8_ crystals (Figure S4).^23^ The B/G ratios estimated from the fluorescence spectra of the crystallized CAS-0.74Tb decreased during the 4 to 8 h heat treatment time while the crystal volume fraction increased (Figure S5). In contrast, there were essentially no changes in the 0 to 4 h range (Figure S5). Notably, there were no detectable changes in the fluorescence intensity ratio after heating the Mo-Free-CAS-0.74Tb at 1000 °C for 9 h, in contrast to the crystallization of the CAS-0.74Tb at 950 °C for 8 h (Figure S6). In addition, the crystal volume fraction determined for the CAS-0.74Tb-8 h specimen indicated that the maximum extent to which metastable CaAl_2_Si_2_O_8_ crystals could precipitate in the CAS glass was 30 vol %. Thus, the glass-phase composition was roughly estimated to be 27CaO–13Al_2_O_3_–60SiO_2_ (wt %) based on subtracting 30 vol % metastable CaAl_2_Si_2_O_8_ crystals from the nominal composition of 25CaO–20Al_2_O_3_–55SiO_2_ (wt %). The fluorescence intensity ratio for the Mo-Free-Al-low-CAS-0.74Tb specimen was essentially the same as that for the Mo-Free-CAS-0.74Tb (Figure S6). It should be noted also that the proportion of Ca^2+^, a network modifier, in the glass composition was changed only minimally when all Al was used to promote crystal precipitation. Specifically, the Ca^2+^ proportion increased from 28.6 to 29.4 mol %. In previous studies, the fluorescence intensity of Tb^3+^ present as a network modifier was found to vary with changes in the amount of the network modifier, implying that the distance between Tb^3+^ ions could affect the fluorescence intensity ratio.^14−19^ In the present study, according to Figures 1 and 2, CAS-1.48Tb displayed an increase in the concentration of metastable CaAl_2_Si_2_O_8_ crystals in a manner similar to that observed in the case of the CAS-0.74Tb (see Figures S3 and S4). In contrast to CAS-0.74Tb, the B/G ratio for the former material decreased with increasing crystal volume fraction as the heat treatment time was varied over the range of 0 to 8 h (Figure 3). Notably, the fluorescence intensities due to electric dipole transitions in the fluorescence spectrum of the CAS-1.48Tb-8 h before normalization were slightly higher than those obtained from the CAS-1.48Tb (Figure 4). The fluorescence intensity at 485 nm due to the ^5^D_4_–^7^F_6_^15^ electric dipole transition was found to increase in addition to an increase in fluorescence because of the ^5^D_4_–^7^F_5_ transition. In addition, the former increase was smaller than the latter. In a previous study,^28^ the intensities of fluorescence peaks related to both the ^5^D_4_–^7^F_5_ and ^5^D_4_–^7^F_6_ transitions increased with increasing Tb^3+^ concentration, and the former increase was larger than the latter. In general, Tb^3+^ luminescence resulting from electric dipole transitions tends to exhibit splitting with decreasing intensity as Tb^3+^ is accommodated into crystals because of an increase in the crystal field.^29,30^

**Figure 5:**
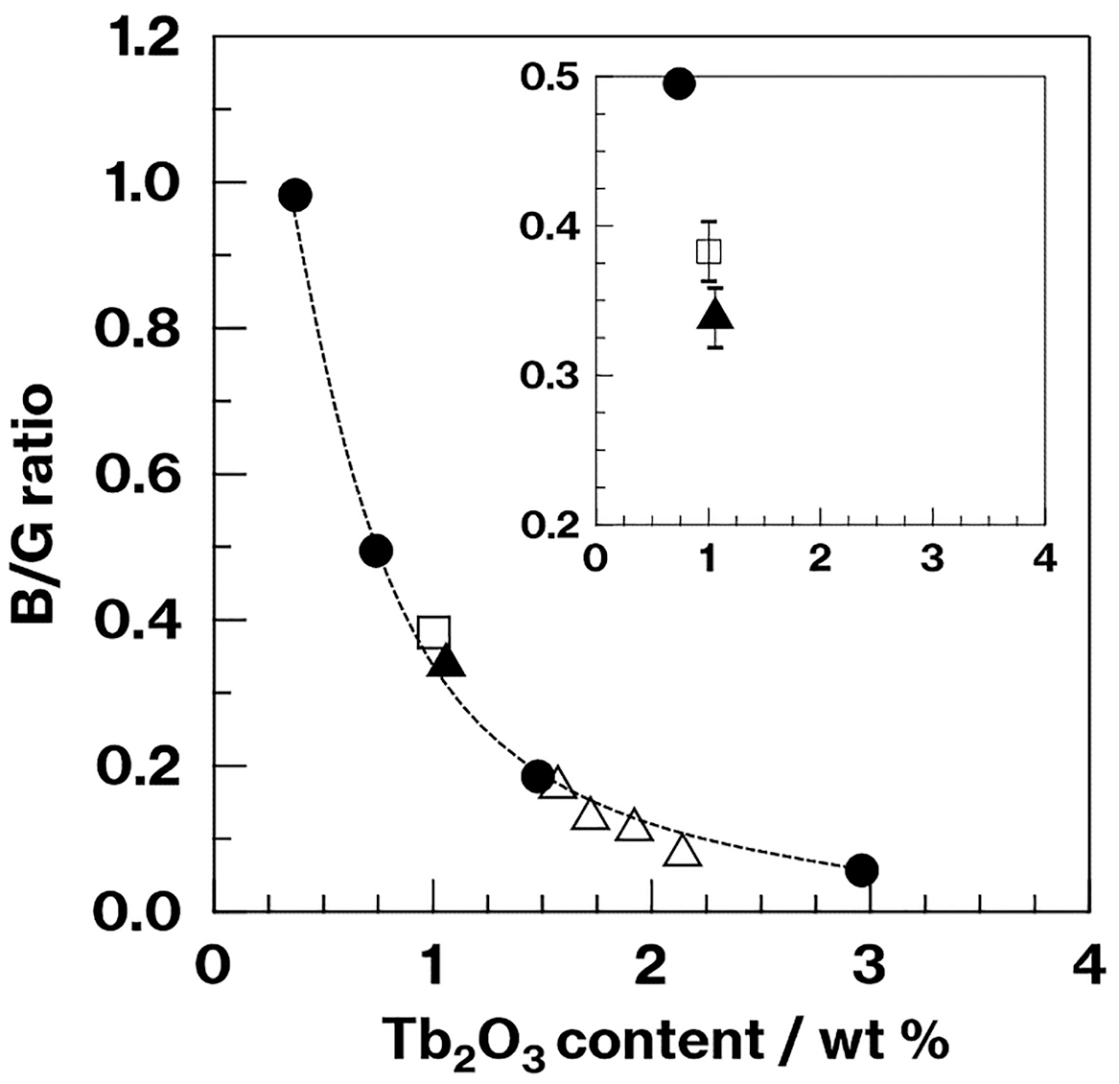
B/G ratios for glass specimens before and after crystallization as functions of the Tb^3+^ concentration. The inset is an enlargement of the 0.5–0.2 B/G ratio range. The dotted line is simply a visual aid. Filled circles represent CAS-0.37, −0.74, 1.48, and −2.96Tb, the filled triangle represents CAS-1.06Tb, and empty triangles and the square represent the crystallized CAS-1.48Tb and CAS-0.74Tb-8 h, respectively.

The data presented above demonstrates that the Tb^3+^ fluorescence intensity ratio was affected by the crystallization of the metastable CaAl_2_Si_2_O_8_ in the CAS glass. Additionally, the intensity ratio change was only minimally dependent on the chemical composition or the heat treatment of the glassy phase. The Tb^3+^ concentration in the glassy phase after crystallization (*C*_A_) could be roughly estimated from the Tb^3+^ concentration before crystallization (*C*_B_) and the crystal volume fraction (*V*) for both the crystallized CAS-0.74 and −1.48Tb (that is, *C*_A_ = *C*_B_ × 100/(100 – *V*)). The resulting values are plotted against the B/G ratios in Figure 5 (filled triangles and empty squares) and show that the relationship between the Tb^3+^ concentration and the B/G ratio was the same as that obtained for the glass containing Tb^3+^ (Figure S3, right).

As an example, the estimated B/G ratio for CAS-0.74Tb-8 h (the open square in Figure 5) with a crystal volume fraction of 30 vol % was close to that for the CAS glass containing 1.06 (0.74 × 100/(100 – 30)) wt % Tb, for which the fluorescence spectrum is provided in Figure S7. According to the relationship between the B/G ratio and Tb^3+^ concentration in Figure 4, the Tb^3+^ concentration increased in the glassy phase with an increase in the proportion of metastable CaAl_2_Si_2_O_8_ crystals. Thus, a decrease in the volume fraction of the glassy phase in which Tb^3+^ ions were concentrated was likely the primary cause of the change in the B/G ratio. Figure S3 shows an increase in the fluorescence intensity because of the ^5^D_4_–^7^F_5_ transition with increasing Tb_2_O_3_ content in the Tb^3+^-doped glass specimens before crystallization. In addition, only a minimal increase in the fluorescence intensity stemming from the ^5^D_4_–^7^F_5_ transition is observed in Figure 4. In the case of the fluorescence spectrum obtained from the CAS-1.48Tb-8 h prior to normalization (Figure 4), the luminescence due to the ^5^D_4_–^7^F_6_ transition appears as very weak shoulders at shorter wavelengths. Recently, Tb^3+^ was successfully accommodated into metastable CaAl_2_Si_2_O_8_, and the resulting Tb^3+^-doped material produced a fluorescence spectrum having a relatively small B/G ratio.^31^ The B/G ratio for the CAS-1.48Tb-8 h in Figure 5 is slightly below the dotted line that shows the relationship between the B/G ratio and Tb_2_O_3_ content as estimated using Tb^3+^-doped glass specimens before crystallization. Thus, the effect of Tb^3+^ accommodation into metastable CaAl_2_Si_2_O_8_ on the B/G ratio is minute when working with the volume fraction range assessed in this study. Therefore, the change in the B/G ratio is mainly dominated by the Tb^3+^ concentration in the glassy phase in the relatively low crystal fraction region. In addition, the effect of Tb^3+^ accommodation into the crystalline phase appears with increasing crystal fraction. Further study will be required to examine changes in the B/G ratio in higher volume fractions to increase the range of application of Tb^3+^ as a probe ion. Such study could allow the analysis of CAS glass with different compositions and crystallinity values^22^ as well as the assessment of other types of glass. Even so, the present study demonstrates that the adjustment of the Tb^3+^ concentration in glass within an optical range was required to successfully probe the extent of crystallization in the case of GC specimens having lower crystal proportions. It should be noted that some of these specimens provided extremely broad XRD profiles (Figures 1 and S4 upper left).

## Conclusions

This study demonstrated the feasibility of using the Tb^3+^ fluorescence intensity ratio to probe the precipitation of metastable CaAl_2_Si_2_O_8_ crystals in CAS glass specimens having relatively low crystal fractions. The results demonstrate that Tb^3+^ luminescence has the potential to act as a versatile probe for glass crystallization after adjusting the Tb^3+^ concentration to the value most appropriate for crystallinity and composition of the glass specimen. In addition, rare-earth ions such as Tb^3+^ can be one of feasible candidates to probe glasses and GCs under a reductive atmosphere. The present technique could also have applications in the field of spectroscopy,^32,33^ where the presence of rare-earth ions in both crystalline and glassy phases with varied compositions might be acceptable and would allow the ready assessment of the amounts of crystalline phases in glassy phases.

## Supporting Information Available

Photographic images of (a) CAS-H and (b) CAS-Eu samples; (c) fluorescence spectrum obtained from CAS-Eu (Figure S1); XRD patterns acquired from CAS-H (upper) and CAS-Eu (lower) (Figure S2); original fluorescence spectra (left) and spectra normalized by 543 nm luminescence intensity (middle) for CAS-0.37Tb, −0.74Tb, −1.48Tb, and −2.96Tb specimens (from the bottom to the top) (Figure S3); XRD patterns obtained from CAS-0.74Tb-2 h, −4 h, and −8 h specimens (upper left, from the bottom to the top) and SEM images of the same materials (upper right and lower) (Figure S4); fluorescence spectra obtained from CAS-0.74Tb, −2, −4, and −8 h normalized by the 543 nm luminescence intensity (left, from the bottom to the top) and the B/G ratios and crystal volume fractions as functions of the heat treatment time (right) (Figure S5); fluorescence spectra obtained from original Mo-Free-CAS-0.74Tb (lower), the same material heated at 1000 °C for 9 h (middle), and Mo-Free-Al-low-CAS-0.74Tb (upper), normalized by the 543 nm luminescence intensity (Figure S6); and fluorescence spectrum obtained from CAS-1.06Tb normalized by the luminescence intensity at 543 nm (Figure S7) (PDF)
