# Supplementary material for: Assessment of the Crystallization Process of CaO–Al2O3–SiO2 Glass Probed with Tb3+ Luminescence
Source: Inorg Chem. 2022 Jul 13;61(29):11478–83. doi: 10.1021/acs.inorgchem.2c01950 (PMC9326970; doi:10.1021/acs.inorgchem.2c01950)
Supplement: Supplementary file 1 — ic2c01950_si_001.pdf [file ic2c01950_si_001.pdf]

Supporting Information

Assessment of crystallization process of $CaO—Al_2O_3—SiO_2$ glass probed with Tb^{3+} luminescence

Shingo Machida, Takuma Yamaguchi, Naoki Emori, Ken-ichi Katsumata, Kei Maeda, Atsuo Yasumori*

Department of Material Science and Technology, Faculty of Advanced Engineering, Tokyo University of Science, 6-3-1 Niijuku, Katsushika-ku, Tokyo 125-8585, Japan

* E-mail: shingo.machida@rs.tus.ac.jp

Figures Supporting Information

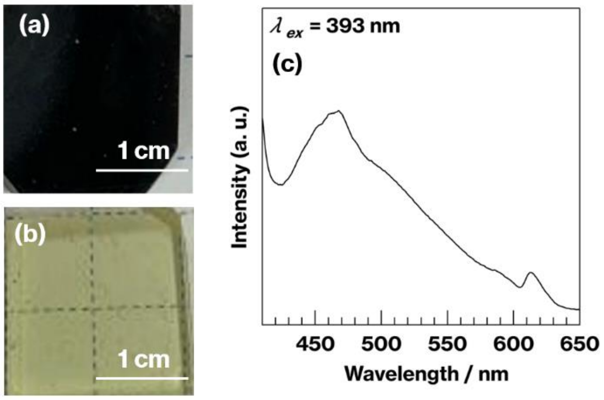

Figure S1. Photographic images of (a) CAS-H and (b) CAS-Eu samples. (c) Fluorescence spectrum obtained from CAS-Eu.

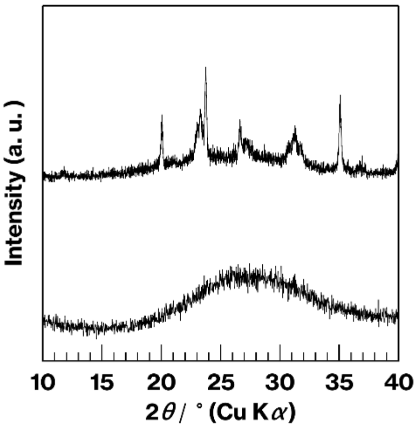

Figure S2. XRD patterns acquired from CAS-H (upper) and CAS-Eu (lower).

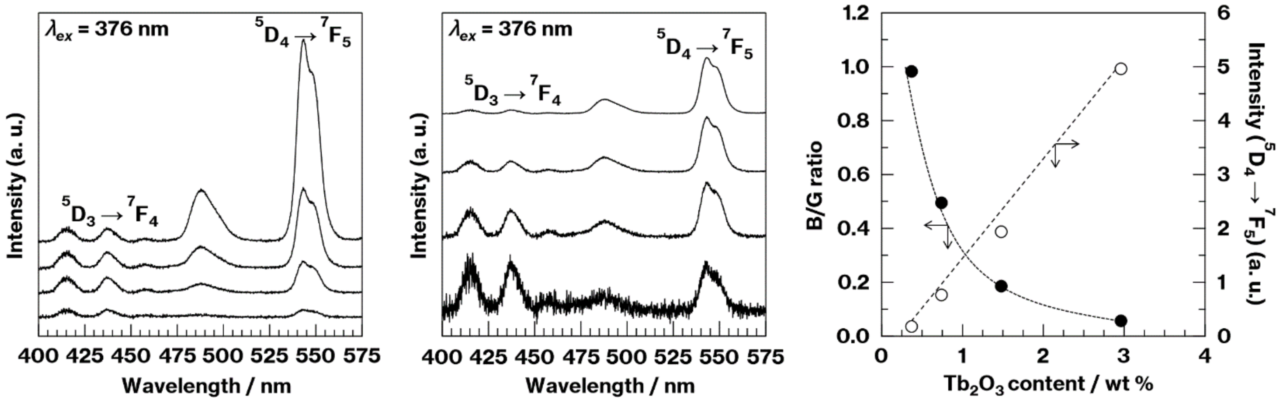

Figure S3. Original fluorescence spectra (left) and spectra normalized by 543 nm luminescence intensity (middle) for CAS-0.37Tb, -0.74Tb, -1.48Tb, and -2.96Tb specimens (from the bottom to the top). B/G ratios and luminescence intensity at 543 nm of CAS glass containing Tb^{3+} as functions of the Tb^{3+} concentration (right). The dotted and dashed lines are simply visual aids. The filled and empty circles represent the B/G ratio and the intensity, respectively.

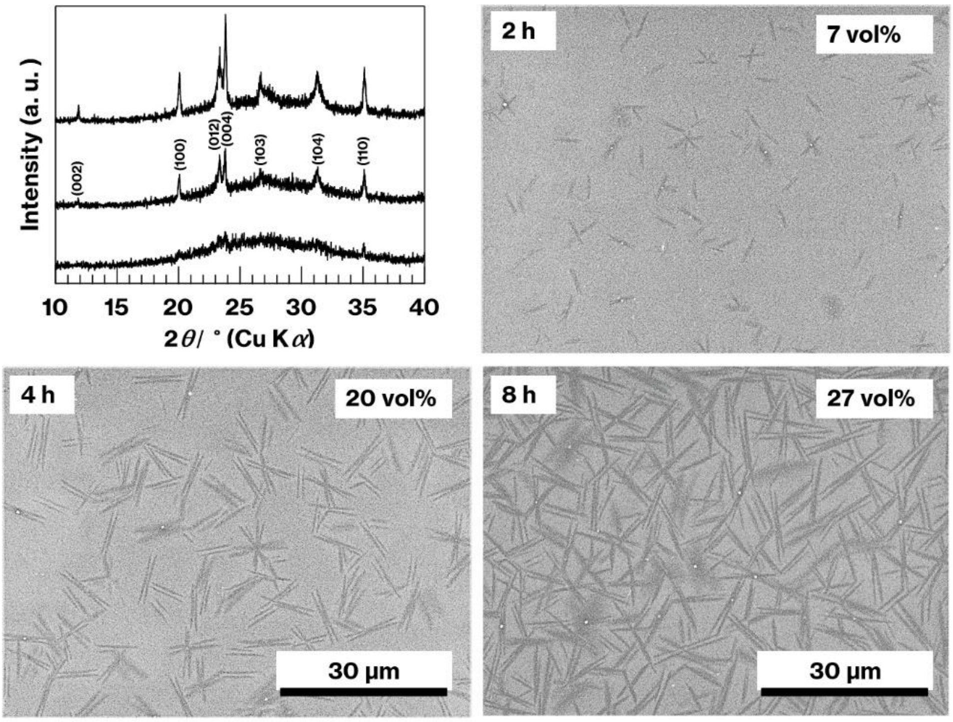

Figure S4. XRD patterns obtained from CAS-0.74Tb-2 h, -4 h, and -8 h specimens (upper left, from the bottom to the top) and SEM images of the same materials (upper right and lower). The heat treatment time and the crystal volume fraction are provided in the upper left and right in each SEM image, respectively.

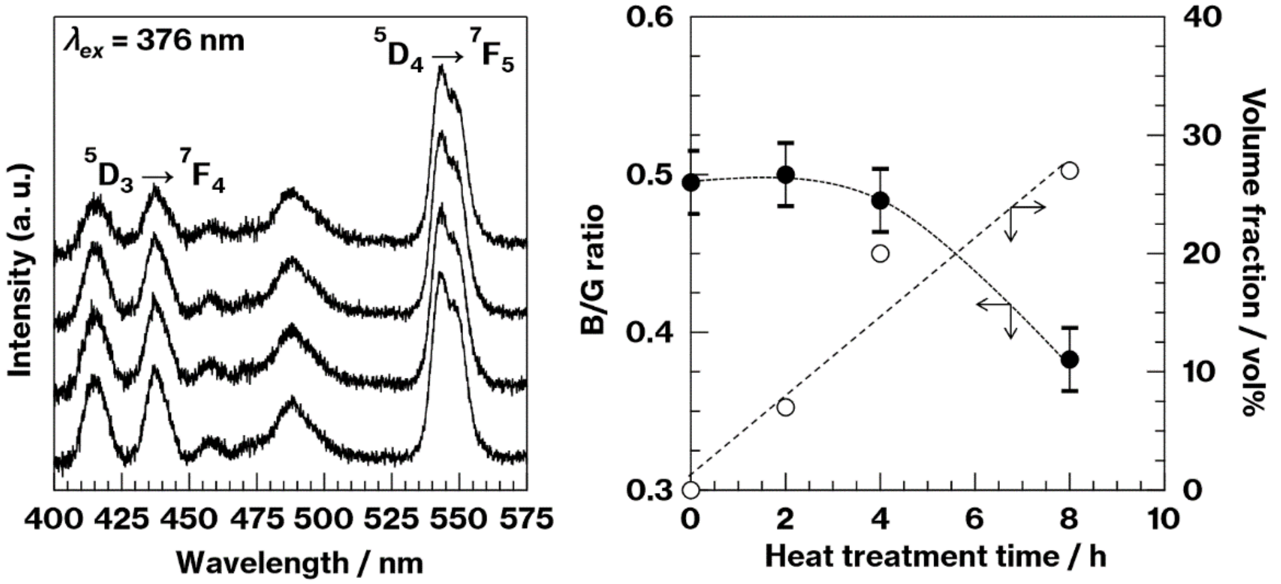

Figure S5. Fluorescence spectra obtained from CAS-0.74Tb, -2 h, -4 h, and -8 h normalized by the 543 nm luminescence intensity (left, from the bottom to the top) and the B/G ratios and crystal volume fractions as functions of the heat treatment time (right). The dotted and dashed lines are simply visual aids. The filled and empty circles represent the B/G ratios and the crystal volume fractions, respectively.

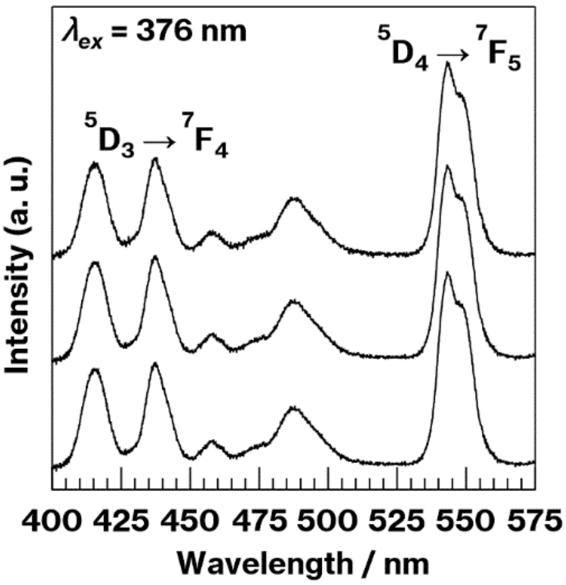

Figure S6. Fluorescence spectra obtained from original Mo-Free-CAS-0.74Tb (lower), the same material heated at 1000 °C for 9 h (middle), and Mo-Free-Al-low-CAS-0.74Tb (upper), normalized by the 543 nm luminescence intensity.

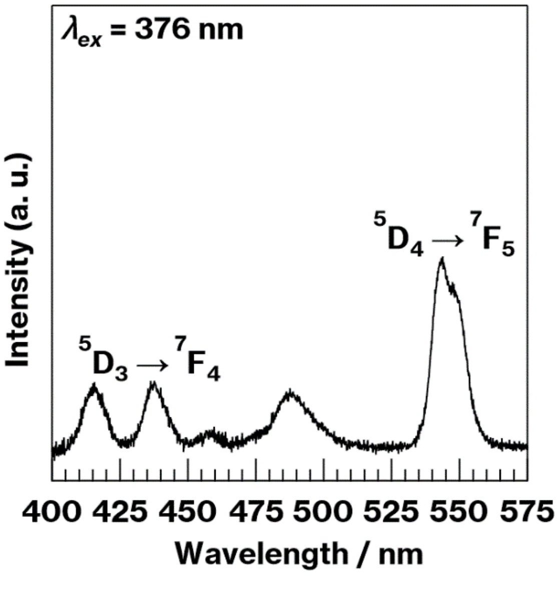

Figure S7. Fluorescence spectrum obtained from CAS-1.06Tb normalized by the luminescence intensity at 543 nm.
